# Antibodies and SARS-CoV-2: New Data on Diagnostics and Therapeutics

**DOI:** 10.3390/life12050614

**Published:** 2022-04-20

**Authors:** Daniele Focosi, Massimo Franchini

**Affiliations:** 1 North-Western Tuscany Blood Bank, Pisa University Hospital, 56124 Pisa, Italy; 2 Department of Hematology and Transfusion Medicine, Carlo Poma Hospital, 46100 Mantova, Italy; massimo.franchini@asst-mantova.it

Welcome to the Special Issue of *Life* entitled *“Neutralizing-Antibody-Based Treatments for COVID-19: Achievements and Lessons Learnt for Future Pandemics”*. The ongoing COVID-19 pandemic has infected nearly 500,000,000 people worldwide, causing more than 6 million deaths and counting. Small-chemical antivirals and immunosuppressive treatments are not always efficacious, but passive (COVID-19 convalescent plasma (CCP) and monoclonal antibodies) and active (vaccines) immunotherapies based on anti-SARS-CoV-2 neutralizing antibodies (nAbs) have proven effective in treating and preventing, respectively, this severe infectious disease at early stages. Among passive immunotherapies, according with the more recent literature evidences CCP has shown a beneficial effect in blocking viral replication and COVID-19 progression when administered properly, i.e., early and at high titer. With the opportunity of being collected and released for clinical use very rapidly and in large amounts, CCP has a clear advantage over the current immunotherapies at following closely the natural evolution of SARS-CoV-2 variants of concern (VOC). This issue is not trivial, considering the continuous virus mutations escaping nAbs, of whatever nature they are (single-nucleotide polymorphisms, deletions, recombinations). In this Special Issue, we will summarize the achievements to date for all treatments based on neutralizing antibodies against COVID-19 and lessons that could prevent errors in future pandemics.

In the first article from Sweden, Marchand and colleagues proposed a quantitative dried blood spot (DBS) test to evaluate the post-vaccination level of nAbs against SARS-CoV-2 [1]. The level of nAbs following vaccination, which have been shown to be correlated to protection from severe diseases, will evolve with time and vary between individuals: Their determination will be fundamental to monitor the degree of individual protection. Several immunoassays quantifying immunoglobulins against the viral Spike (S) protein in serum/plasma have been developed, but the need for venous blood samples could limit the frequency and scale of control in large populations. In this context, the use of a self-collected, quantitative DBS, which simplifies such monitoring, appears particularly attractive. The authors investigated this new method, which combined DBS and an anti-SARS-CoV-2 immunoassay, in two studies. The first study investigated 14 volunteers who received two doses of the BNT162b2 (Comirnaty^®^, Pfizer/BioNTech, New York, NY, USA and Mainz, Germany) mRNA vaccine. The anti-S antibodies were highly stimulated by the second dose and peaked two weeks later. The antibody levels subsequently decreased, and 3 months later, they were down to 65%. The second cohort was composed of 200 randomly selected Swedish patients, with no information on previous COVID-19 infections or vaccination, of whom 87% of the subjects had detectable anti-S immunoglobulins. In both cohorts, DBS proved to be sufficiently sensitive for evaluating the immune status against SARS-CoV-2. 

In the second article from Italy, Focosi and colleagues investigated the cross-reactivity between SARS-CoV-2 and other Coronaviridae and showed that IgG against nucleocapsid protein of alphacoronavirus NL63 and 229E correlate with a maximal World Health Organization’s (WHO) clinical severity score ≥5 in hospitalized COVID-19 patients (incidence rate ratios was 1.87 and 1.80, respectively, and 1.94 for the combination) [2]. Altogether, these laboratory findings suggest a possible role of previous alphacoronavirus immunity on the severity of SARS-CoV-2 infection through an antibody-dependent enhancement (ADE)-like mechanism.

In the third article from Poland, Malecki and colleagues reported a series of 13 pediatric patients hospitalized for COVID-19 and receiving CCP therapy [3]. The median hospitalization time was 20 days while the median post-CCP virus elimination time was 6 days. All were fragile patients with at least one pre-existing co-morbidity. Notably, clinical improvement was achieved in all patients with no adverse effects were found in any of the cases. Based on these interesting results, the investigators concluded that CCP could be a promising treatment for COVID-19 in children.

The fourth article, by Moubarak and colleagues, addresses the issue of the continuous emergence of more virulent SARS-CoV-2 variants with increasingly high basic reproductive numbers [4]. Strategies aimed at mitigating the deleterious effect of successive waves of COVID-19 include primarily more effective vaccination strategies with coverage of greater parts of populations. From a treatment viewpoint, the use of CCP collected recently and specifically directed against SARS-CoV-2 variants of concern (VOC) can increase survival rates and improves host responses to viral challenge. 

Finally, the last article by Franchini and colleagues summarizes the more recent clinical evidences on the use of CCP [5]. The authors identified three factors as key determinants of CCP efficacy: the treatment (CCP), the disease (COVID-19) and the patients. After a careful analysis of the published literature the investigators pointed out high-titer and transfusion as close to symptom onset as possible as the main predictors of effectiveness of CCP. In addition, immunocompromised COVID-19 patients, still unable to mount a protective immune response against SARS-CoV-2 after multiple vaccine dose, are those who benefit most from this passive immunotherapy.

In conclusion, the articles reported here demonstrate how important antibody-based therapies against COVID-19 are and that their clinical and laboratory research should continue with the aim of optimizing their clinical use.
